# Spying on small wildlife sounds using affordable collar-mounted miniature microphones: an innovative method to record individual daylong vocalisations in chipmunks

**DOI:** 10.1038/srep10118

**Published:** 2015-05-06

**Authors:** Charline Couchoux, Maxime Aubert, Dany Garant, Denis Réale

**Affiliations:** 1 Département des Sciences Biologiques, Université du Québec à Montréal, H3C 3P8 Montréal, QC, Canada; 2 Département de Biologie, Université de Sherbrooke, J1K 2R1, Sherbrooke, QC, Canada

## Abstract

Technological advances can greatly benefit the scientific community by making new areas of research accessible. The study of animal vocal communication, in particular, can gain new insights and knowledge from technological improvements in recording equipment. Our comprehension of the acoustic signals emitted by animals would be greatly improved if we could continuously track the daily natural emissions of individuals in the wild, especially in the context of integrating individual variation into evolutionary ecology research questions. We show here how this can be accomplished using an operational tiny audio recorder that can easily be fitted as an on-board acoustic data-logger on small free-ranging animals. The high-quality 24 h acoustic recording logged on the spy microphone device allowed us to very efficiently collect daylong chipmunk vocalisations, giving us much more detailed data than the classical use of a directional microphone over an entire field season. The recordings also allowed us to monitor individual activity patterns and record incredibly long resting heart rates, and to identify self-scratching events and even whining from pre-emerging pups in their maternal burrow.

New research questions become increasingly accessible through technological breakthroughs, particularly for the study of animal vocal communication. In the past few years, for instance, the development and successful use of new bioacoustic technologies based on microphone arrays have allowed researchers to record the sounds produced by wildlife and relate this to their ecology and habitat^1^,^2^. Since most ecologists cannot, unfortunately, turn themselves into micro-electrotechnicians, it appears most interesting to monitor the continuous development of cutting-edge devices in areas distant from our field of expertise, as sometimes an inventive but simple deviation of an apparatus from its primary purpose (see for instance the ingenious use of “burrowscopes”^3^) can make a significant difference for an entire field of research. Miniaturisation of the equipment, in particular, makes it more and more possible to observe undisturbed animal behaviour in detail for multiple individuals^4^. Indeed, whereas non-invasive methods that are performed from a remote location^1^,^2^ are preferred whenever possible, it becomes necessary to equip the animal with a monitoring device when research questions are based at the individual level.

This is particularly interesting considering the generalised growing interest in individual differences^5^ that have led scientists to rethink and adapt their approach in many research areas. In the field of evolutionary ecology, there is thus a need to better integrate and understand among-individual differences in behavioural tendencies within natural populations (i.e. animal personality^6^,^7^,^8^). For instance, the study of vocal communication systems would highly benefit from focusing on among-individual differences, which can be monitored through on-board acoustic data-loggers. However, thus far such devices have been developed to fit specific species characteristics (marine mammals^9^ or bats^10^), and they are either too big to be used on small terrestrial animals^11^,^12^ or they are not readily available for deployment in the wild^13^.

To overcome these limitations, we suggest using an “off-the-shelf” professional miniaturised audio recorder device that is commercially available (approx. 300 $) and that can be easily and readily used to monitor free-ranging small animal sound emissions for 24 continuous hours at a time (frequency range from 100 to 10,000 Hz). The Edic-mini Tiny A31 model (http://ts-market.com/products/models/1198/), the world’s smallest digital audio recorder (weight = 6 g; *Guinness World Records* holder), is developed by the Russian company TS-Market (http://www.ts-market.com/) specialised in the design and the manufacture of professional miniature audio and video devices. Through a simple re-packaging with a lighter protective encasing (i.e. shape-adapted), we were able to design a system mounted into a collar to fit small animals (eastern chipmunks, *Tamias striatus*) weighing 100 g or more (Fig. 1, Supplementary Fig. S1 and Methods). The technological advances made in the world of espionage thus allow to spy on the vocal emissions of individuals from natural populations.

In this methodological study, we show the validity and the efficiency (in terms of both quantity and quality) of this affordable, operational and robust miniaturised audio recorder device compared to the classical use of a directional microphone for recording emissions from focal individuals. We also present several types of research questions that could be addressed using the spy microphones.

## Results

### Validity and efficiency of recording with spy microphones

The spy microphone devices proved to be extremely robust and wild-proof since they were retrieved without any damage, despite being subject to rough conditions by chipmunks that spend part of their time underground in the narrow tunnels of the burrow.

The number of calls recorded at different times of the day with the spy microphones reflected the number of calls recorded throughout the field season with the directional microphone (Fig. 2a, Spearman rank-correlation test: ρ = 0.80, S = 56.56, P = 0.002, n = 10). We also found a positive relationship between the proportion of the different types of calls recorded with the two methods, although the small period over which the spy microphones have been used and the small number of call types prevented us from finding a significant association between the two methods (see details on the types of calls in ref 14; Fig. 2b). This indicates that the spy microphones could easily replace more standard techniques for some research protocols in bioacoustics.

Spy microphones were highly efficient at recording calls while human observers were absent from the area, and their recording performances became even more compelling when considering the reduced recording effort needed to obtain the data. They allowed us to record 450 calls during the first 12 h of recording for the 9 equipped individuals. In comparison, surveying the same 9 individuals with classical methodology (directional microphone) for 176 h throughout the field season allowed us to record manually only 250 of their calls. Since our study was performed on chipmunks that are solitary and therefore emit calls to their conspecifics at a distance, we are highly confident that we could differentiate the calls emitted by the collared individuals from those emitted by conspecifics, especially since the sound captured at the throat of the animal was extremely loud. Studies in which individuals tend to aggregate to vocalise might first need to measure the loudness of the sounds expected at standardised distances through tests and observations (see also the use of a specific device for this purpose in ref 13).

Using spy microphones therefore increased the sample size by a factor of 3.43 ± 2.30 (mean ± SD) for each of the studied individuals (Fig. 3). Furthermore, in terms of sampling effort, we recorded 4.35 ± 3.98 (mean ± SD) calls per hour with spy microphones compared to only 0.16 ± 0.13 per hour with the directional recording technique (paired Wilcoxon signed rank test, V = 2, P = 0.012, n = 9), and calls recorded with the spy microphone represented 80.91 ± 32.74% (mean ± SD) of all the calls collected for each individual.

With spy microphones, the recording is captured on the throat of the animal, which produces clear signals that sound like those recorded in lab conditions (Fig. 4). Ideally, the frequency of the sound recorded should not exceed 11 kHz so that all of the acoustic characteristics of the sounds can be retrieved (the maximum sampling frequency is 22 kHz as it was designed to record human voice, see the manual for further details on the acoustic characteristics of the device).

### Beyond vocalisations: individual heart rate, activity patterns, self-scratching events and whining

The 24 h continuous recording allowed by the spy microphone device also revealed other sounds that could be identified and analysed in different research contexts. Spy microphones could be particularly well suited to monitoring the activity and time budget of individuals^12^. In chipmunks, above ground foraging activities would induce more noisy recordings compare to periods of relative inactivity (e.g. an individual resting in its burrow) and this variation in decibel level can be quantified through variation in grey intensity in the spectrogram (Fig. 5a; measured with the software ImageJ^15^). Activity patterns can then be estimated at different periods of the day to compare the behaviour of different individuals (Fig. 5b) or to estimate the mean activity level for a population (Fig. 5c). The activity budget could also be established in even more detail, as we can easily identify other sounds on a spectrogram (e.g. self-scratching events, Supplementary Fig. S2a, b). Resting heart rates can also be measured through the recordings. These were mostly accessible during the night portion of the recording, but several were also obtained during the day, when chipmunks appeared to be resting (Supplementary Fig. S2c). Other interesting sounds are also recognisable, such as whining that seemed to be emitted by pre-emerging pups in the burrow of their mother (Supplementary Fig. S2d, Supplementary Audio A1).

## Discussion

The simultaneous recording of several individuals allowed by this method represents an obvious advantage for efficient data recording. This technique is especially appealing to record the emissions from individuals that are difficult to observe/detect in the field or for those located at the edges of a study area, making it easier to sample the complete range of individual behaviours. It will also allow a more detailed investigation of the information encoded in the calls by facilitating repeated recordings of vocalisations. Equipping each individual several times during the season would allow one to acquire a sufficient quantity of calls to investigate intra- and inter-session variation in the vocalisations.

The spy microphones also recorded a wider spectrum of the chipmunks’ call diversity than the traditional technique. The more natural conditions in which the recordings are made with this method allowed us to record several “chip” calls, which are usually hard to record when following focal individuals, as they emit “trill” calls in response to human presence (Fig. 2b). Recording continuous individual behaviour could thus allow one to record vocalisations that are rarely elicited and therefore obtain a better measure of the complete vocal repertoire of the studied species.

Sounds recorded with the spy microphones are extremely pure and can therefore be easily analysed for acoustic measurements, and such quality could be particularly interesting for researchers interested in harmonics. The quality of recording also provides surrounding sounds with a good quality, which could then permit the determination of the soundscapes of an animal’s environment^1^ (see Supplementary Fig. S3 for examples of bird songs recorded and Supplementary Audio A2 - sound of Canadian geese).

The daylong recordings performed by the on-board devices could also be used to monitor individual activities in detail^12^. This technique would allow researchers interested in time budgets to monitor individuals simultaneously and efficiently, especially for small animals that are difficult to track continuously in the field.

During the inactive phases of the individuals, the quality of the recordings produced exceptional data as we were able to obtain resting heart rate measurements without the more common highly invasive techniques such as surgery and implantation of monitors^16^,^17^. These can be used as actual resting heart rate to compare them with heart rate under stress to measure different processes such as coping styles^18^.

The quantity of self-scratches that occurred throughout the day for the equipped individuals could be used to estimate their parasite load, as previous studies showed that grooming behaviours are related to ectoparasite loads^19^,^20^.

The recordings retrieved from the devices set on females before pup emergence all contained whining, which clearly seemed to be emitted by pups in the maternal burrow. These sounds could be useful to assess maternal care, mother-offspring relationship or to study the ontogeny of the calls in species where pups are inaccessible before emergence from burrows.

Obviously, the spy microphones could also be used for bigger animals in concert with some other devices such as accelerometers^13^, GPS or radio transmitters^12^ to gather complementary data along with the recording of sounds.

In summary, these spy microphones are highly efficient tiny audio recorders that are readily available and can be easily used as on-board acoustic data loggers to survey the behaviour of free-ranging small animals.

## Methods

### Setting the on-animal device on chipmunks

The collar-mounted devices were successfully fitted and recovered on 21 chipmunks, during summers 2013 (5) and 2014 (respectively 9 and 7 on two different study sites), as part of a long-term project that investigates the evolutionary ecology of this species in a deciduous forest in Southern Québec, Canada.

Chipmunks in this population regularly wear collars for a long period (they are equipped with collar-mounted radio transmitters to find their burrows and thermo-sensors to monitor their torpor activity during winter^21^) but the spy microphone device is a bit bigger and heavier (5.5 g vs 2.2–4.1 g for the other devices, which represents 4.9–6.2% of the body mass of the chipmunks tested). We thus retrieved the device as soon as the recording was finished to limit the impact on chipmunks. We put the collars on as many individuals as possible during a day, then did not return to the site for the next 24 hours to ensure our presence did not affect the recordings. We returned to the site on the third day to capture the individuals and recover the devices. When active above ground chipmunks can be easily captured, which allowed us to limit the time that they spent with the collar. Fitting a collar only takes a minute for an experienced handler and does not require any anaesthesia. After the handling, we monitored the behaviour of the equipped individuals to ensure that they supported the device and that they returned to their natural activities, such as foraging or grooming, until they reached their burrow (see examples of individuals equipped with the device and that performed such natural behaviours in Supplementary Videos V1a,b, V2 & V3). This monitoring also allowed us to make sure that individuals had safely reached their burrow if they needed further time to recover. To increase our probability of retrieving the collars on the third day, we performed targeted trapping at the individual burrows and added Sherman traps that are bigger than the Longworth traps (30.5 × 9.5 × 7.5 cm vs 14 × 6.5 × 8.5 cm) and that individuals equipped with collars seem to prefer.

All captures and manipulations were carried out in accordance with the guidelines of the Canadian Council on Animal Care and the protocols were approved through the Université du Québec à Montréal (permit CIPA #0512-760-0513) and the Québec Ministry of Sustainable Development, Environment, Wildlife and Parks (permit SEG #2014-04-22-103-05-S-F).

Spy microphones can also be used to record vocalisations when they are not set on chipmunks, as they are easier to handle than a directional shotgun microphone plus a recorder. With this method of recording sounds are not missed by delays in pressing start/stop on the recorder and there is no need to change batteries, which would be the case for daylong recording using traditional methods.

### Audio recording analyses

**Figure 1:**
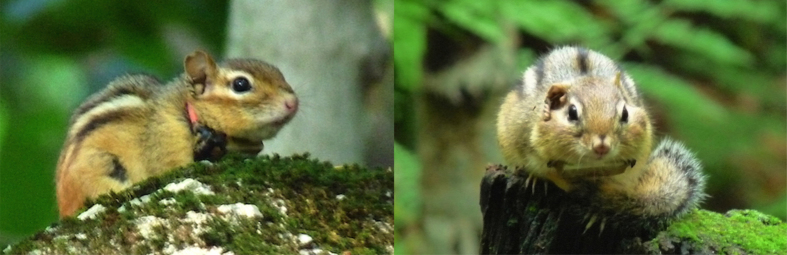
**A collar-mounted spy microphone for recording individual sound emissions in free-ranging chipmunks.** Two individual chipmunks equipped with the device for a recording of 24 h in natural conditions. Photos by C. Couchoux.

**Figure 2:**
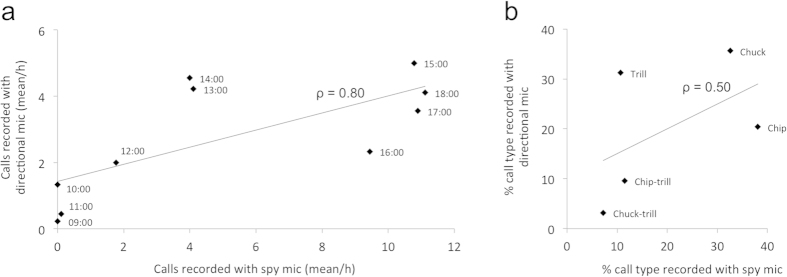
**Test of the validity of the recording method: recording data are similar for the recordings made with the spy microphones (24 h) and with the classic manual recording (directional microphone; entire field season).** (**a**) Comparison of the mean number of calls recorded through the two techniques, considering the time of the day (the different points represent the mean number of calls recorded at different times of the day, for the corresponding hour). (**b**) Comparison of the percentage of call types recorded using the two techniques. Mean number of calls and percentage of call types were calculated based on the emissions of nine individuals followed during the field season and equipped with the spy microphones on the same day and at the same site.

**Figure 3:**
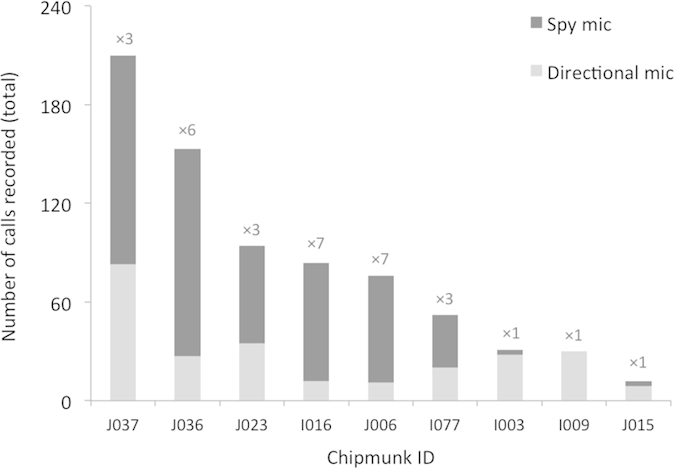
**Representation of the spy microphone’s high efficiency to record a great quantity of calls, by comparison to the quantity of calls recorded through the directional microphone.** Illustration of the raw number of calls (without considering the sampling effort) recorded with the two techniques, showing the high proportion of the sample size due to calls recorded with the spy microphones. Numbers above each bar indicate the increase in individual sample size allowed with the recordings from the spy microphones.

**Figure 4:**
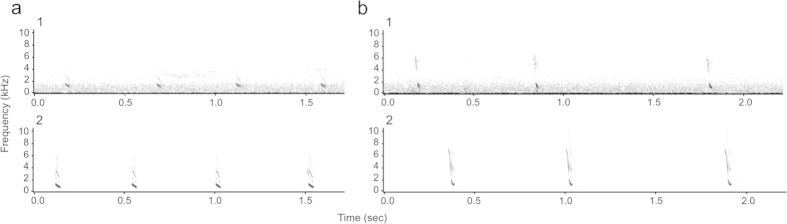
**Sound spectrograms of low frequency “chuck” calls emitted in a bout, illustrating the high quality of recordings obtained from spy microphones.** (**a**,**b**) Comparison of four “chuck’ calls recorded for two different individuals (**a** and **b**) with the directional microphone (**a1** and **b1**, recordings chosen for being of high quality) and with the spy microphones (**a2** and **b2**, raw recordings not submitted to any filter). The grey scale represents the intensity of the sounds, so that louder sounds appear darker. The sounds recorded with the directional microphone contain a certain level of background noise (fuzzy lighter grey in spectrograms **a1** and **b1**, mainly caused by windy conditions), which overlaps with the calls and could affect the acoustic measurements, whereas the vocalisations recorded with the spy microphones are very clean and not affected by interference noise.

**Figure 5:**
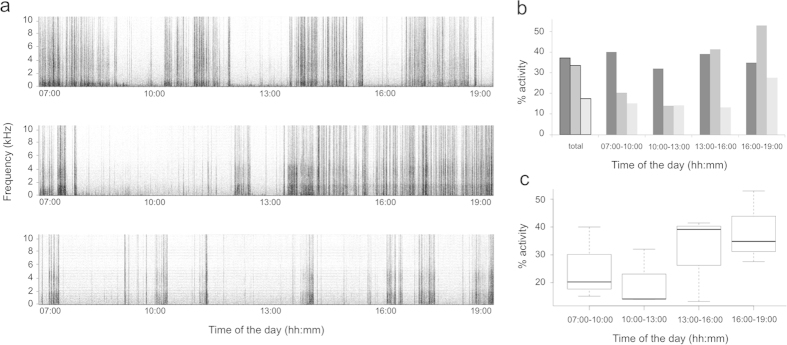
**Illustration of the potential to monitor individual activity levels through the use of noises produced by and around the chipmunk.** (**a**) Large view spectrograms representing activity patterns of three different individual chipmunks between 7:00 am and 7:00 pm. (**b**) The activity levels measured and compared for the three individuals illustrated in (**a**) (individuals from top to bottom in (**a**) are represented from left to right in (**b**) from darker to lighter grey bars), for the total 12 h of recording and for each separated period of 3 h. The percentage of activity for different times of the day is estimated based on grey intensity indices measured on the spectrograms (the percentage is calculated as the value of grey intensity for the measured period divided by the maximum value of intensity possible (for black)). (**c**) Population activity levels at different periods of the day (here based on the values estimated in b, for the three individuals).

We present here the data from the recordings done on nine eastern chipmunks that were equipped with the device (Fig. 1; see Supplementary Fig. S1 for further illustrations of the device), on the same day (22 June 2014) and at the same site. The first 12 h (7:00 am–7:00 pm) of the 24 h audio recordings were visually inspected for identification of the sounds recorded (software AVISOFT-SAS Lab Pro, through a 10 seconds view, and FFT-length = 1024).

The validity and the efficiency of recording with the spy microphones were assessed through a comparison with the recordings made on the same individuals throughout the field season using a shotgun directional microphone (Sennheiser ME67/K6 connected to a Marantz PMD661 portable recorder; on 23 days, from 6 June to 20 August 2014, 176 hours of presence on the site for recording). Non-parametric correlations tests (for small sample sizes) were implemented to compare the efficiency of recording between the two methods.

### Spy microphone specificities & settings

The recorder has several acoustic features and recording options that can be found in the operational manual (http://ts-market.com/upload/iblock/db4/Operational%20Manual%20Tiny.pdf). We chose the 600 h rechargeable version of the device that allows the maximum amount of recording time. The programming of the device is quite straightforward, being done through a firmware accessible on the device once the USB connection is established. The recording settings were chosen to record with the highest quality and capacity of recording, by choosing a recording format without any compression and selecting the highest sampling frequency (22 kHz).

When retrieving the recordings, done using the USB connection, sections can be chosen for downloading. For a 24 h recording, two separate files of 12 h must be downloaded. The battery can be recharged in a few hours.

We chose to use the timer recording option to schedule the 24 h recording at 7:00 am the day after the capture (after synchronising the clock on the device with that of the computer), to ensure that the chipmunks had recovered from the manipulation and to therefore obtain meaningful behavioural data. There is also a VAS mode available on the device that records only those sounds that are above a defined threshold.

### Design of the wild-proof collar-mounted spy microphone

In order to mount the spy microphone into a collar that could be fitted on chipmunks (Fig. 1), we removed the heavy rectangular-shaped metal case (Supplementary Fig. S1a_**1,2**_) that protects the circuit (containing the data logger and the microphone) and battery (Fig. S1a_2-4_, circuit: green and black part, battery: silver part). For this purpose, the device can be ordered unglued in the case, allowing you to fashion it your own way. After carefully removing the device from the case (Fig. S1a), the tiny metal spring (Fig. S1a_3,4_) was cautiously removed as its only purpose is to hold the device inside the case. The little white square pad that is attached to the battery (Fig. S1a_4_, visible on the left part of the battery) was left in place as it insulates the battery from the circuit to avoid a short-circuit. There is also a tiny white switch above the plug connection (Fig. S1a_,1,3,4_) that can be used to turn the recording on and off. For our purpose we only used the programming function of the recorder so the switch was always set on the ‘off’ position (switch set on the left, closest to the microphone) and it was no longer accessible once assembled. The device was then set in a light protective case made of a large tube of heat-shrink (Fig. S1b, the heat-shrink is the transparent envelope (_**1**_) that after being heated slightly with a flame takes the shape of the material it covers (_**2-4**_)). The heat-shrink was cautiously sealed at the extremities (Fig. S1b_2-4_) by pressing the warm open sides of the tube with tweezers (Fig. S1b_2_) so that dust, earth, or water could not enter the device. Then some openings were created (with a box cutter) in front of the plug connection and the microphone (Fig. S1b_3_, result seen in Fig. S1d_3_). To individually identify the spy microphones (crucial for unmistakable simultaneous setting and recovery of the different devices on multiple individuals), a label bearing the device number was placed on top of the heat-shrink encased device (Fig. S1c_1,_). Figure S1c shows how the collar was then mounted using another (transparent) wide heat-shrink tube to encase the device (_**1**_) and to hold a smaller heat-shrink tube (black) that allowed the passage of a ty-rap (_**2**_), that served as the collar. The shape of the collar (that fits the study animal’s neck) was obtained by curving the (black) heat-shrink tube (again with heat, Fig. S1c_3_) and by stuffing FIMO (green matter in Fig. S1c_4_) in-between the device and the (black) heat-shrink tube receiving the ty-rap. The holes were also sealed to prevent the animal from grabbing the device. The shape of the collar was adapted to suit the animal’s neck by simply fastening the extremities of the ty-rap together (with the green wire, Fig. S1d_1-4_). Two additional heat-shrink tubes (red) were added on each side of the ty-rap to protect the animal’s neck (Fig. S1d_1-5_).

Finally, the open extremities of the second large heat-shrink (that stayed open with the FIMO, Fig. S1d_1_) were covered with resistant tape (black electrical tape, Fig. S1d_2-5_). The holes made in front of the microphone and the plug were left open, to allow programming using the USB cable (Fig. S1d_3_). Figure S1d_3_ shows the final version of the collar-mounted spy microphone that could then be used successively on different chipmunks. The entire device weighed 5.5 g. Once the device was programmed, a supplementary piece of tape was added to cover the microphone and plug entrances (Fig. S1d_4_). The device was then ready to use (Fig. S1d_5_) on a free-ranging animal. Once the device was retrieved we remove the tape in front of the plug openings to download the data (Fig. S1d_3_). Before fitting the device on the next animal, the ty-rap was changed and a new piece of tape was placed to cover the plug (Fig. S1d_4_). The device could be shaped differently to fit the requirements of other species (see e.g. in ref^13^).

## Additional Information

**How to cite this article**: Couchoux, C. *et al*. Spying on small wildlife sounds using affordable collar-mounted miniature microphones: an innovative method to record individual daylong vocalisations in chipmunks. *Sci. Rep.* **5**, 10118; doi: 10.1038/srep10118 (2015).

## Supplementary Material

Supplementary Information

Supplementary Audio Recording A1

Supplementary Audio Recording A2

Supplementary Video V1

Supplementary Video V2

Supplementary Video V3

Supplementary Video V4
