# Supplementary material for: Spying on small wildlife sounds using affordable collar-mounted miniature microphones: an innovative method to record individual daylong vocalisations in chipmunks
Source: Sci Rep. 2015 May 6;5:10118. doi: 10.1038/srep10118 (PMC4650754; doi:10.1038/srep10118)
Supplement: Supplementary Information [file srep10118-s1.pdf]

Supplementary information

Spying on small wildlife sounds using affordable collar-mounted miniature microphones: an innovative method to record daylong vocalisations, tested on chipmunks.

Charline Couchoux[1,*], Maxime Aubert[1], Dany Garant[2] & Denis Réale[1]

[1] Département des Sciences Biologiques, Université du Québec à Montréal, H3C 3P8 Montréal, QC, Canada
[2] Département de Biologie, Université de Sherbrooke, J1K 2R1, Sherbrooke, QC, Canada

*Correspondence should be addressed to C.C. (charline5@msn.com).

Supplementary Figures

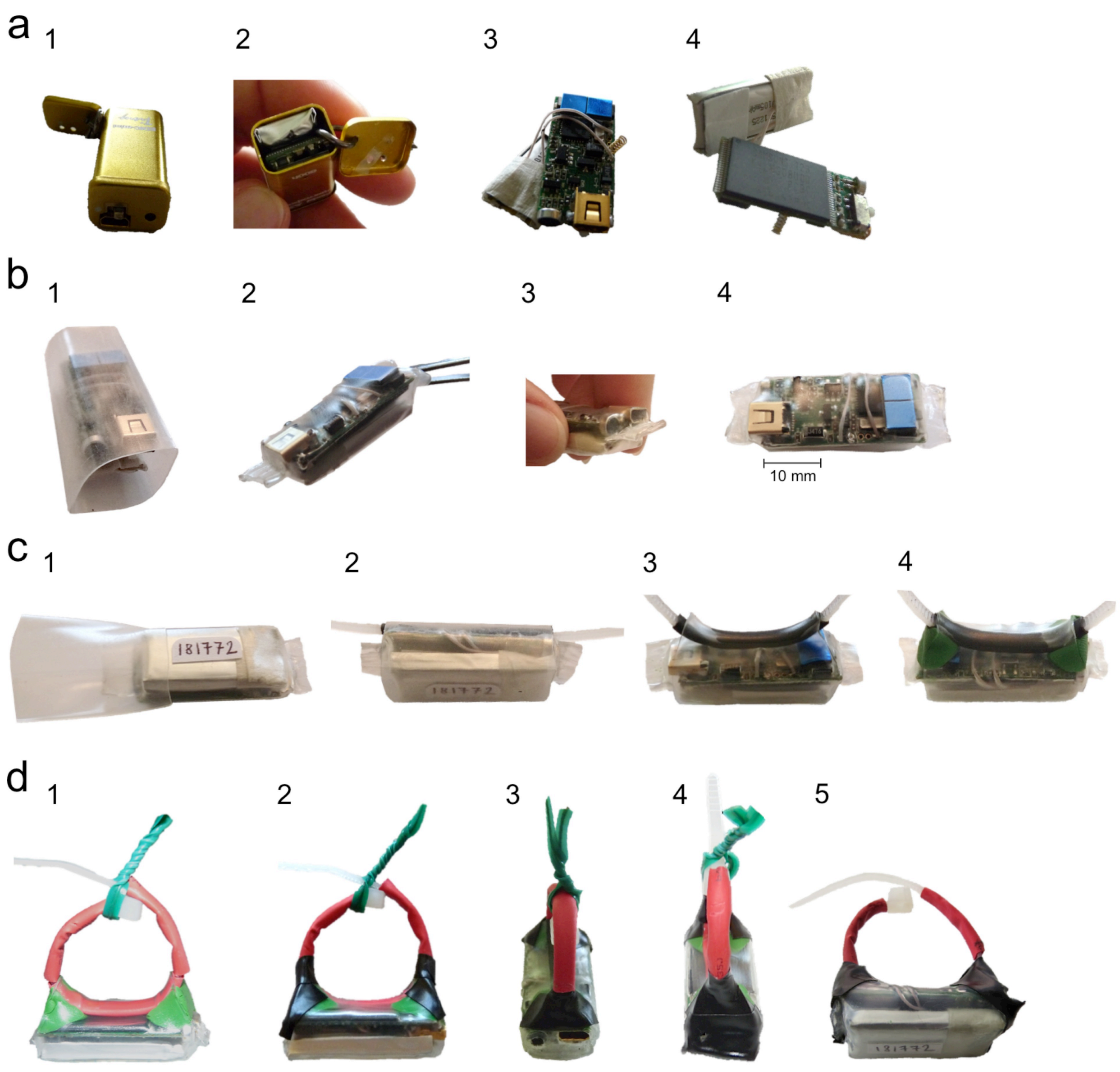

Supplementary Figure S1: Illustration of the different steps implemented to design the spy microphone device into a wearable collar-mounted acoustic data logger for small mammals (details in the Methods section).

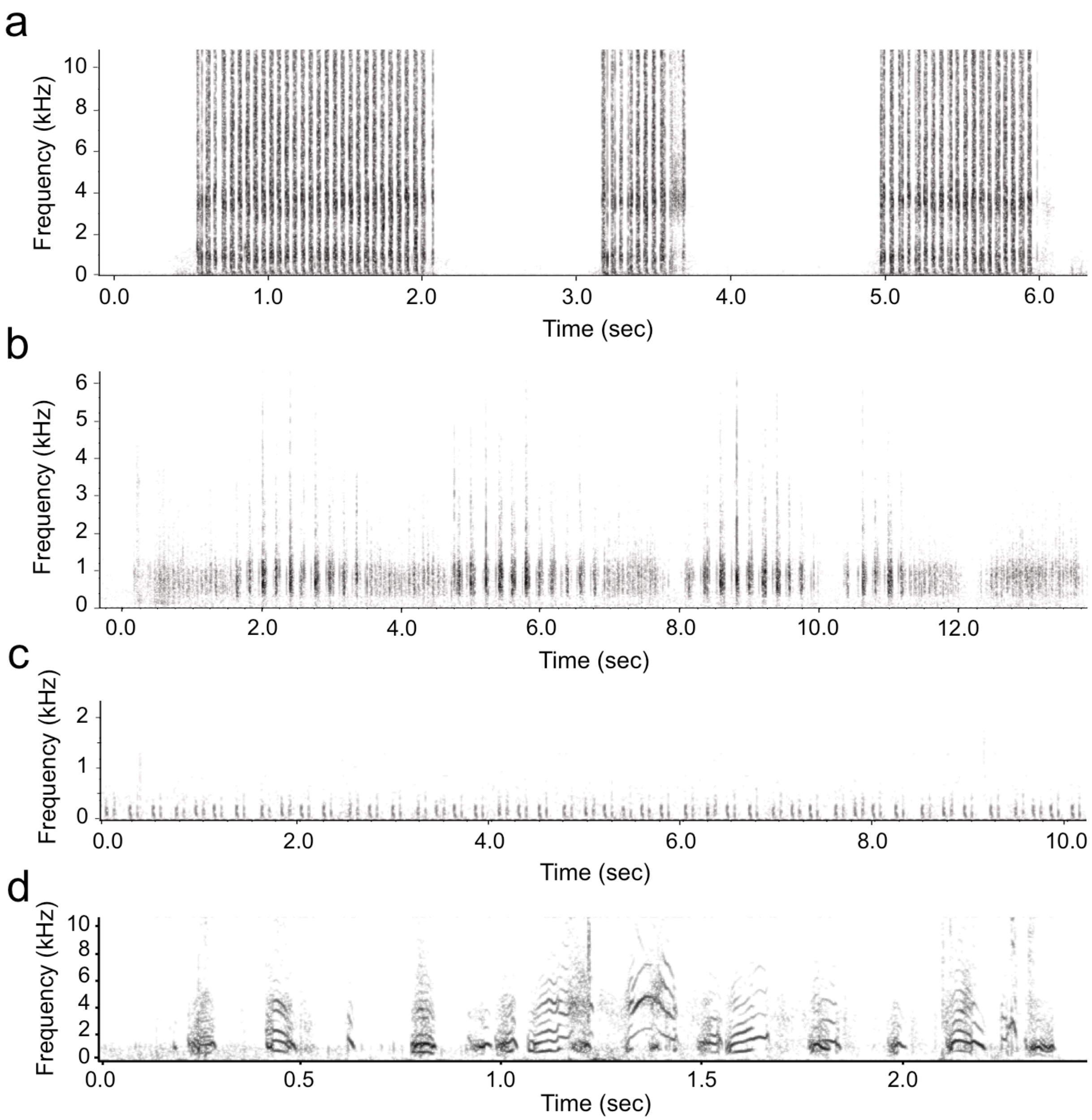

Supplementary Figure S2: Sound spectrograms showing different behavioural data that were obtained with the spy microphone recordings. Illustration of (**a,b**) two sequences of self-scratching, (**c**) a high quality resting heart rate and (**d**) whining recorded in a female burrow before pup emergence.

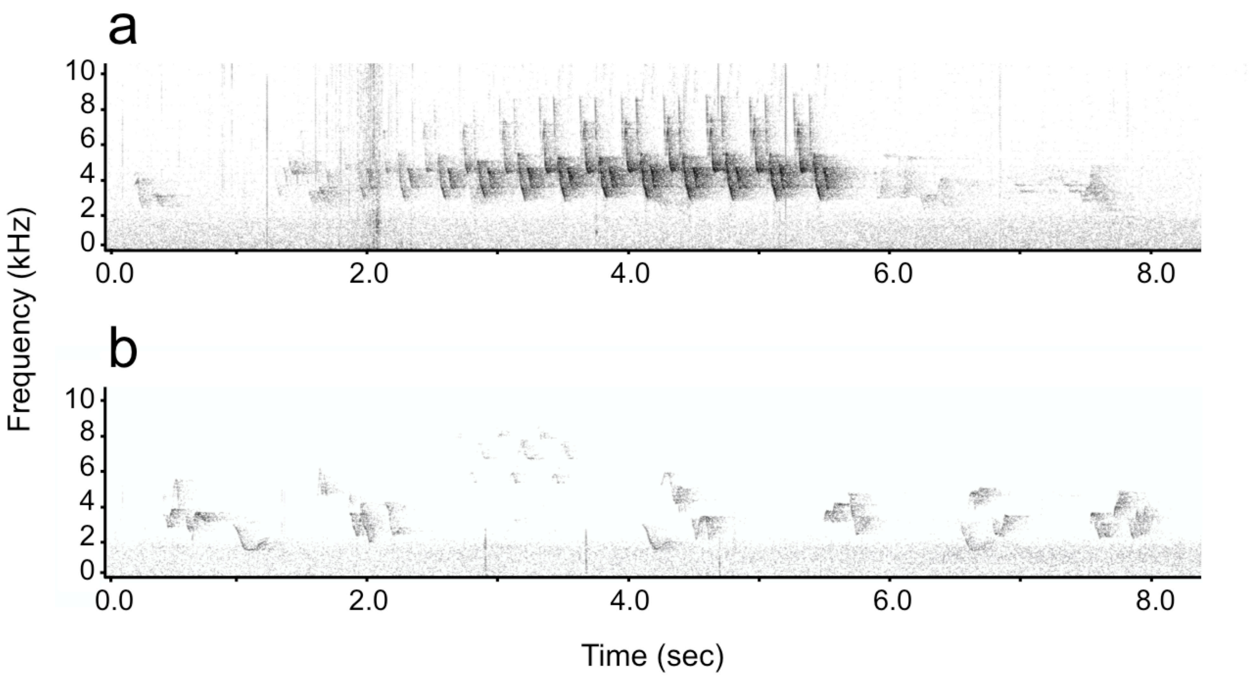

Supplementary Figure S3: Sound spectrograms illustrating the possibility of recording the soundscapes of the individuals equipped with the spy microphone. Vocalisations of (**a**) an ovenbird (*Seiurus aurocapilla)* and (**b**) a red-eyed vireo (*Vireo olivaceus)*.

Supplementary Audio and Video files

Supplementary Audio Recording A1: Example of whining recorded by a female chipmunk equipped with the spy microphone, sounding like emissions from pre-emerging pups in the maternal burrow.

Supplementary Audio Recording A2: Illustration of the soundscape recorded by a chipmunk equipped with the spy microphone: sounds of Canadian geese passing flying over the canopy.

Supplementary Video V1: Example of an individual chipmunk equipped with the spy microphone, attending to its natural activities such as (**a**) feeding on a beechnut and (**b**) grooming.

Supplementary Video V2: Example of an individual chipmunk equipped with the spy microphone, attending to its natural activities such as foraging and self-scratching under windy conditions.

Supplementary Video V3: Example of an individual chipmunk equipped with the spy microphone, entering its burrow.
